# Investigation on the Failure Behavior of Engineered Cementitious Composites under Freeze-Thaw Cycles

**DOI:** 10.3390/ma12111808

**Published:** 2019-06-04

**Authors:** Junfei Zhong, Jun Shi, Jiyang Shen, Guangchun Zhou, Zonglin Wang

**Affiliations:** 1 School of Transportation Science and Engineering, Harbin Institute of Technology, Harbin 150090, China; Stefan_Chung@outlook.com (J.Z.); wangzonglin@vip.163.com (Z.W.); 2 Key Lab of Structures Dynamic Behavior and Control of the Ministry of Education, Harbin Institute of Technology, Harbin 150090, China; 18S133127@stu.hit.edu.cn (J.S.); gzhou@hit.edu.cn (G.Z.); 3 Key Lab of Smart Prevention and Mitigation of Civil Engineering Disasters of the Ministry of Industry and Information Technology, Harbin Institute of Technology, Harbin 150090, China

**Keywords:** engineered cementitious composites, freeze-thaw cycles, fiber reinforcement, frost-induced failure, three-level evaluation mode, Taguchi method

## Abstract

This paper investigates the freeze-thaw performance of engineered cementitious composites (ECC) reinforced with polyvinyl alcohol (PVA) fibers, by applying an innovative criterion for judging the specimen’s working state mutation. The ECC materials are prepared into 25 mixtures using the Taguchi method. Then, the fundamental transverse frequency, the flexural performance and the internal strain variation of ECC specimens subjected to freeze-thaw cycles are measured. Unlike the existing studies, this investigation focuses on the failure behavior of ECC materials in the process of freeze-thaw. The Mann-Kendall (M-K) criterion is introduced to detect the ECC specimen’s working state leap feature, leading to the updated definition of frost-induced failure concept. Furthermore, the three-level model for evaluating the freeze-thaw performance of ECC materials is established according to the revealed essential leap feature. Thus, the effect of each individual mix design factor on the frost-induced failure indices is perceived from the signal-to-noise (S/N) ratio analysis and the analysis of variance (ANOVA). Finally, a mix formulation estimated based on Taguchi method is recommended for its optimum resistance against frost-induced failure, which is verified by the confirmation experiment.

## 1. Introduction

The brittleness, low tensile strain strength and inadequate extensibility are among the drawbacks of ordinary cement-based materials. Recent efforts to increase the ductility and toughness of such materials have led to the development of fiber reinforced concrete (FRC) [1], ultra-high-performance concrete (UHPC) [2] and engineered cementitious composites (ECC) [3,4,5,6]. The ECC is generally prepared by incorporating the reinforcement of randomly distributed high-performance fibers among which polyvinyl alcohol (PVA) fiber provides a desirable balance of performance and cost for improving the ductility, toughness and other performance. Many studies reported that the special surface property and strong fiber-matrix bond make PVA fibers of competitive merits versus other fiber reinforcements [7,8,9]. The substantial benefit of using PVA fibers was obtained by controlling the crack width and raising the ultimate tensile strain up to 3~7% [10]. Strain hardening could be induced as the distinctive characteristic of ECC to encourage distributed energy dissipation across the volume of structural systems. Another important component adopted in the ECC mixture is the fly ash (partial substitution of cement), with the purpose of economizing resource and enhancing the mechanical performance of ECC [11,12,13]. The mode in which fly ash affects the ECC could be explained as the modification of porosity characteristics and the interface with aggregates brought about by this type of mineral admixture.

The utilization of PVA fibers and fly ash help to strengthen the ECC performance subjected to mechanical load. Moreover, the severe environmental conditions influencing ECC in the long term should also be considered. One of the aggressive environmental hazards is the cyclic freezing and thawing, which is fairly common in cold regions like the northeast of China. During the freeze-thaw cycles, the water-ice phase change and heat mitigation could induce strain in the matrix. Accordingly, the total strain during freeze-thaw cycles is composed of freezing-induced strain and thermal strain. These strains feature either expansion or contraction behavior depending on the moisture content of the specimen [14]. Besides, the permeation of dissolved deicing salts during the thawing period is bound to cause further corrosion which lowers the service life and occasionally leads to unpredictable failure of ECC materials. The addition of PVA fibers into ECC mixtures is indicated to improve frost resistance by entraining air voids inside the matrix [15,16]. Additionally, PVA fibers have the capacity to control the width of cracks and mitigate the spalling of the cementitious matrix under increasing freeze-thaw cycles [17]. These advantages of PVA fibers facilitate the ECC to experience less reduction in the dynamic modulus of elasticity and mechanical properties. On the other hand, the microstructure tailored by fly ash results in the decrease of capillary pores that could restrict the access for free water. However, it should be noted that the presence of fly ash probably hinders the frost resistance of cement-based materials because of its slow setting actions and strength development under frost exposure [18,19,20,21]. It is recommended that the fineness and content of fly ash, as well as the water-to-binder ratio should be carefully determined depending on the specific use of cement-based materials.

This investigation incorporates the PVA fibers and fly ash into the cementitious materials targeting to obtain the ECC mix formulations which provide desirable resistance to freeze-thaw damage. The freeze-thaw resistance is normally evaluated based on the loss in some particular engineering properties, such as mass, dynamic modulus of elastic and mechanical strength after a certain number of freeze-thaw cycles [15,16,22,23]. The terminal state is emphasized by the present literature which largely ignores the analysis on the overall freeze-thaw process to delve the working behavior of materials. As the freeze-thaw action is going on, the evolving loss in engineering property expresses the quantitative change whose development naturally produces the qualitative change of working behavior. Differing from the conventional freeze-thaw test, this investigation applies the maximum cycles on the ECC materials to obtain not only the ultimate freeze-thaw bearing capacity, but also the whole freeze-thaw state. With the support of Mann-Kendall (M-K) method, the working behavior of ECC materials is characterized and the updated frost-induced failure is identified according to the natural law from quantitative change to qualitative change. Moreover, the influence of PVA fibers and fly ash on the resistance to frost-induced failure are studied and the mix design formulation is optimized. These efforts constitute an interesting supplement to the current knowledge of freeze-thaw behavior characteristics of ECC materials. Consequently, the ECC materials designed referring to the revealed characteristics will be more reasonable to be applied to the engineering projects in the cold regions. 

## 2. Experimental Work 

### 2.1. Materials

**Figure 1:**
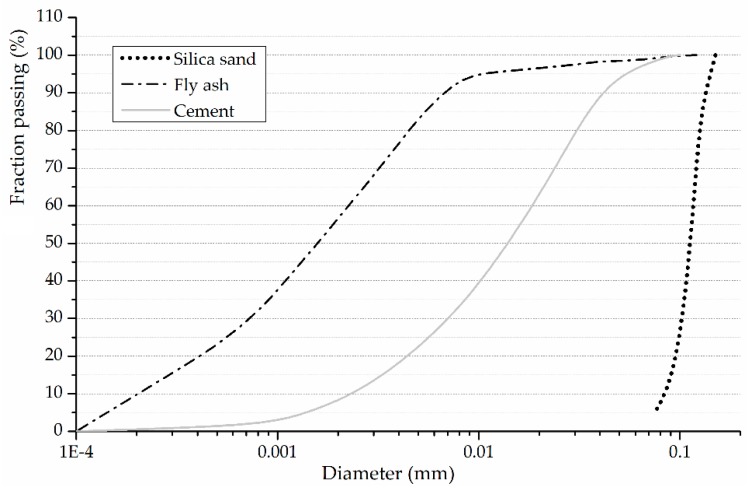
The particle size distributions of P.O. 42.5 type cement, ground fly ash and silica sand.

**Figure 2:**
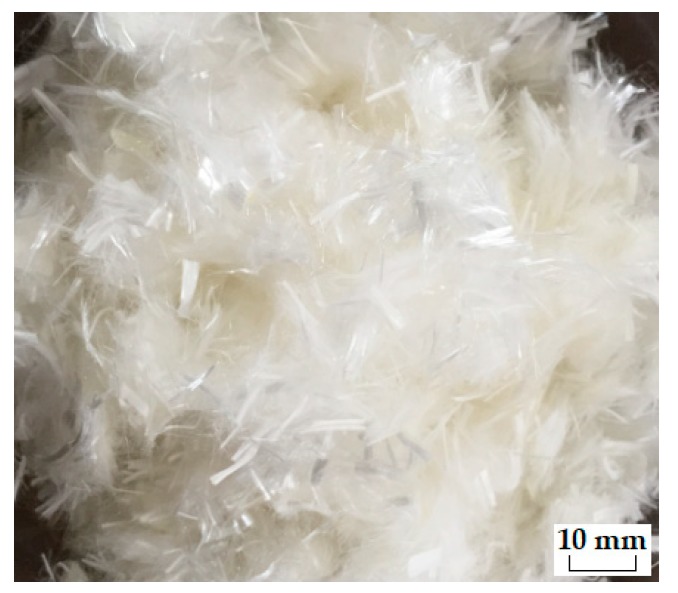
The PVA fibers used in this investigation.

In this investigation, the P.O. 42.5 type cement and fly ash from the local thermoelectric plant are both used as the cementitious binders. The milling apparatus is used to grind original fly ash into much finer ones, which is found to be more reactive in pozzolanic effect at a relatively early age [24,25]. The chemical compositions of P.O. 42.5 type cement and the ground fly ash are summarized in Table 1 and Figure 1 presents their particle size distribution. The silica sand with the maximum particle diameter of 0.150 mm is used as the fine aggregates and the particle size distribution is also presented in Figure 1. Polycarboxylic superplasticizer is added in the ECC mixtures to produce a desired fresh mix workability. The reinforcement materials for ECC are the PVA fibers manufactured by Kuraray Co., Ltd (Tokyo, Japan), as shown in Figure 2. Table 2 presents the properties of PVA fibers. 

### 2.2. Mix Design Method

**Figure 3:**
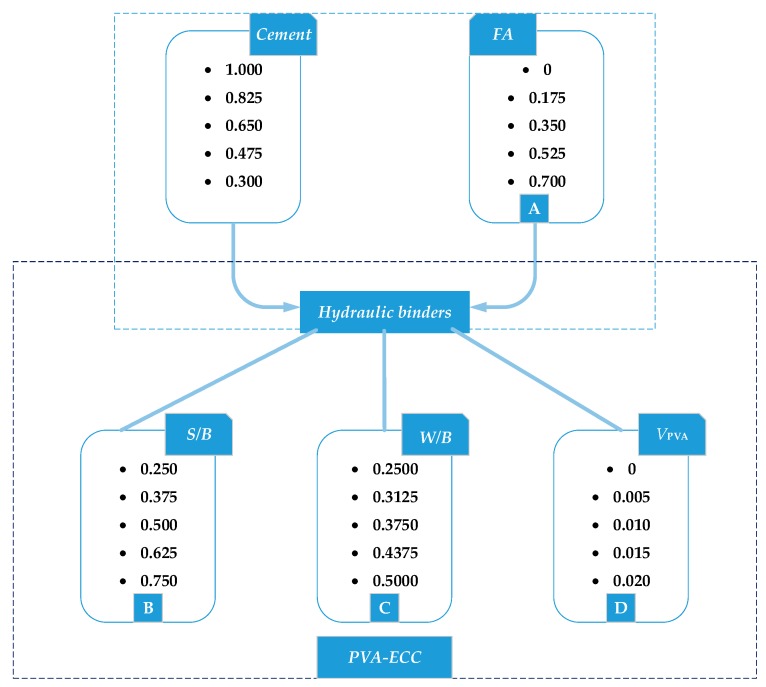
The schematic diagram of varying levels for mix design factors.

The ground fly ash, silica sand, Portland cement, fibers and water were blended proportionally to produce the PVA fiber reinforced ECC materials. The advanced mix design techniques concerning multiple variables were applied based on the Taguchi method, which is particularly efficient in studying the influence of multi-variables when compared with the exhaustive test of every possible trial [26,27,28,29]. The mix design factors were selected as: Fly ash content (*FA*, by weight), sand-to-binder ratio (*S*/*B*), water-to-binder ratio (*W*/*B*) and the volume fraction of PVA fiber (*V_PVA_*). Basically, the more levels provide the Taguchi-based experiment with higher accuracy, the greater cost of raw materials. In order to balance effectiveness and cost, five typical levels were assigned to each factor, as shown in Figure 3. The 625 (= 5^4^) combinations of these mix design parameters were reduced to 25 mix formulations using the *L*_25_ orthogonal array proposed by Taguchi method, as summarized in Table 3. The mass fraction of superplasticizer added for each mix formulation is also presented in Table 3. 

### 2.3. Experimental Methods

**Figure 4:**
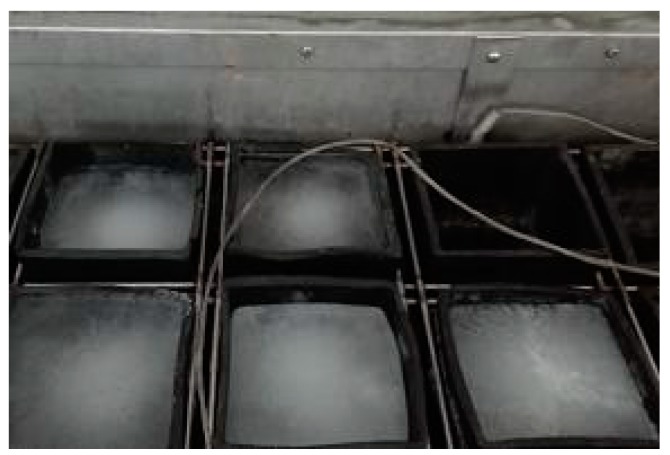
Specimens placed in the rubber sleeves.

Rapidly repeated cycles of freezing and thawing were performed on prismatic specimens with the size of 100 mm × 100 mm × 400 mm per GB/T 50082-2009 [30] and ASTM C666 [31]. Specimens were cured for 24 d at 20 ± 2 °C and 95% relative humidity, immediately followed by the vacuum water-saturation for 4 d. The freezing-and-thawing apparatus (TDR-1) was used and the specimens were submerged in specified rubber sleeves filled with water, as shown in Figure 4. The refrigerating and heating procedures were controlled to produce continuously, automatically, and reproducible cycles. Thermocouples were placed inside the control specimen and the thermal exchange fluid to obtain the central temperature which the ECC material undergoes, as well as its surrounding temperature. The highest temperature at the center of the specimen shall be −18 ± 2 °C, and the lowest temperature shall be 4 ± 2 °C.

**Figure 5:**
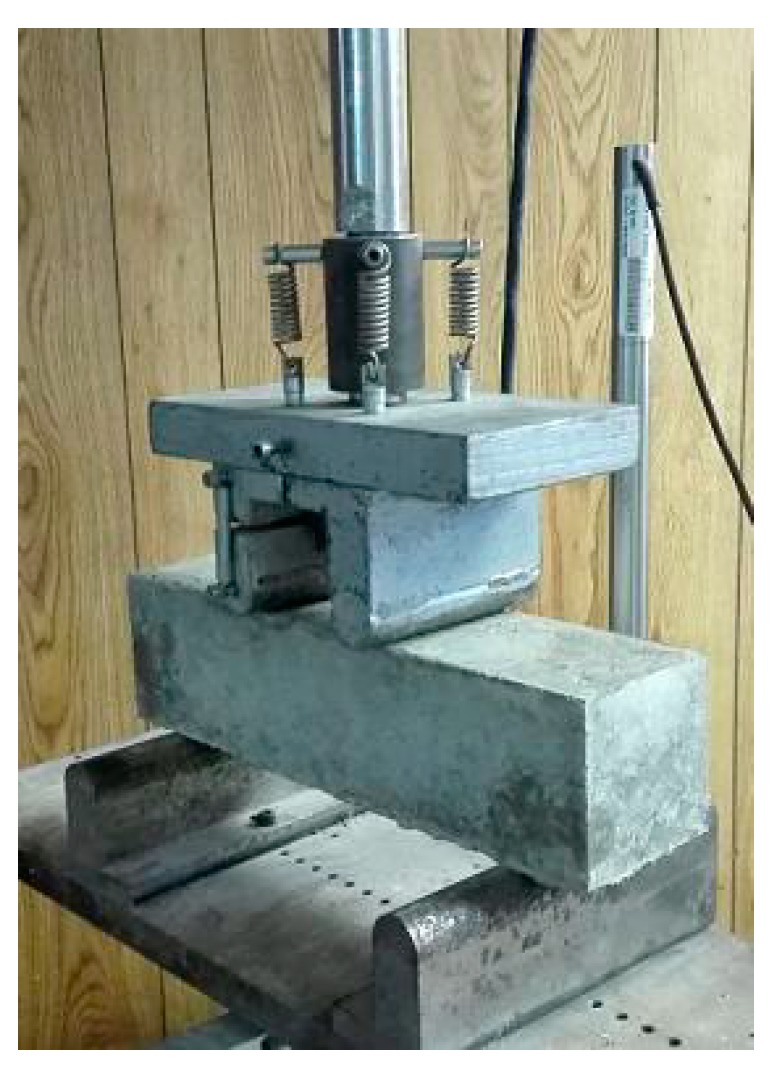
The flexural strength test setup.

Specimens were exposed to cyclic freeze-thaw action till the final degradation, in order to obtain their ultimate freeze-thaw bearing capacity. The fundamental transverse frequency test was conducted to detect the internal damage after every 25 cycles, where the dynamic modulus of elasticity (DME) was calculated per GB/T 50082-2009 [30] and ASTM C666 [31]. The flexural strength is an indispensable performance characteristic for the ductile ECC materials and the prism-shaped specimens for the flexure test are also suitable for the freeze-thaw test. The loss in flexural strength has been verified by existing literature to be effective in reflecting the frost damage as well [15,16]. It should be a reasonable choice to measure the flexural strength of ECC specimens under freeze-thaw cycles. To this effect, the flexure test was performed before the freeze-thaw test and also after every 25 cycles using a sevovalve-controlled hydraulic test system operated at a displacement rate of 0.02 mm/sec on prismatic specimens via four-point loading (Figure 5).

**Figure 6:**
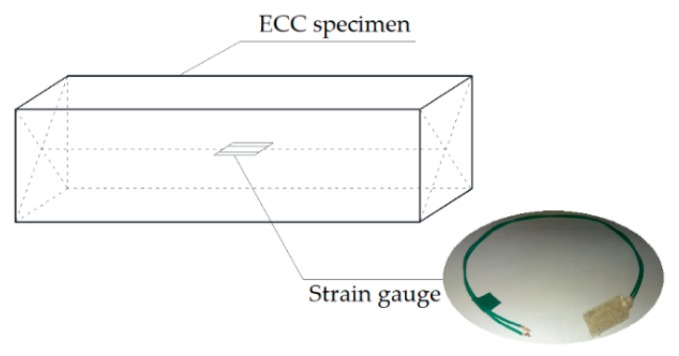
The location of strain gauge in the ECC specimen.

Besides, the real-time strains inside the ECC specimens were continuously monitored. The strain gauge covered with plastic sheets was embedded in the center of additional ECC specimen for each mixture, as shown in Figure 6. The strain gauges were connected to the DH3816 automatic static strain data-logger, the data acquisition rate of which was set as once every 10 min. The actual strain is temperature compensated by deducting the strain of the dried specimen measured in the same condition.

## 3. Results and Analysis

### 3.1. Flexural Load-Deflection Curves

**Figure 7:**
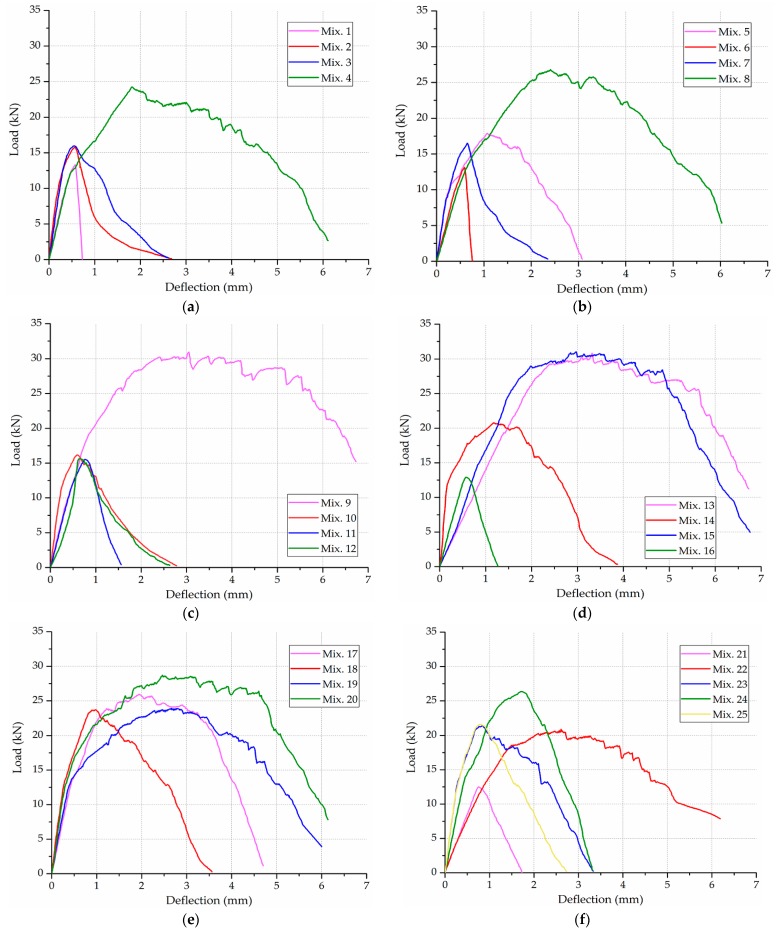
The flexural load-deflection curves before the freeze-thaw test for: (**a**) Mix. 1~4; (**b**) Mix. 5~8; (**c**) Mix. 9~12; (**d**) Mix. 13~16; (**e**) Mix. 17~20; (**f**) Mix. 21~25.

The flexural load-deflection curves before the freeze-thaw test for all the ECC mixtures are illustrated in Figure 7.

The load-deflection curve varies in shape according to the set of mix design. Generally, the fibrous ECC specimens (e.g., Mix. 9, 13 and 15) exhibit superior load capacity and post-cracking performance to the plain specimens (e.g., Mix. 1, 6 and 11). The larger area under the load-deflection curve up to a specified deflection for the specimen reinforced with PVA fiber indicates its better energy dissipation capacity. The peak load is discerned from the load-deflection data to calculate the maximum flexural stress, which is equivalent to the flexural strength discussed in this investigation. The effect of mix design factors on the flexural strength and the extent to which they affect this performance characteristic are analyzed in subsequent sections.

### 3.2. The Freeze-Thaw Working State of ECC Mixtures

**Figure 8:**
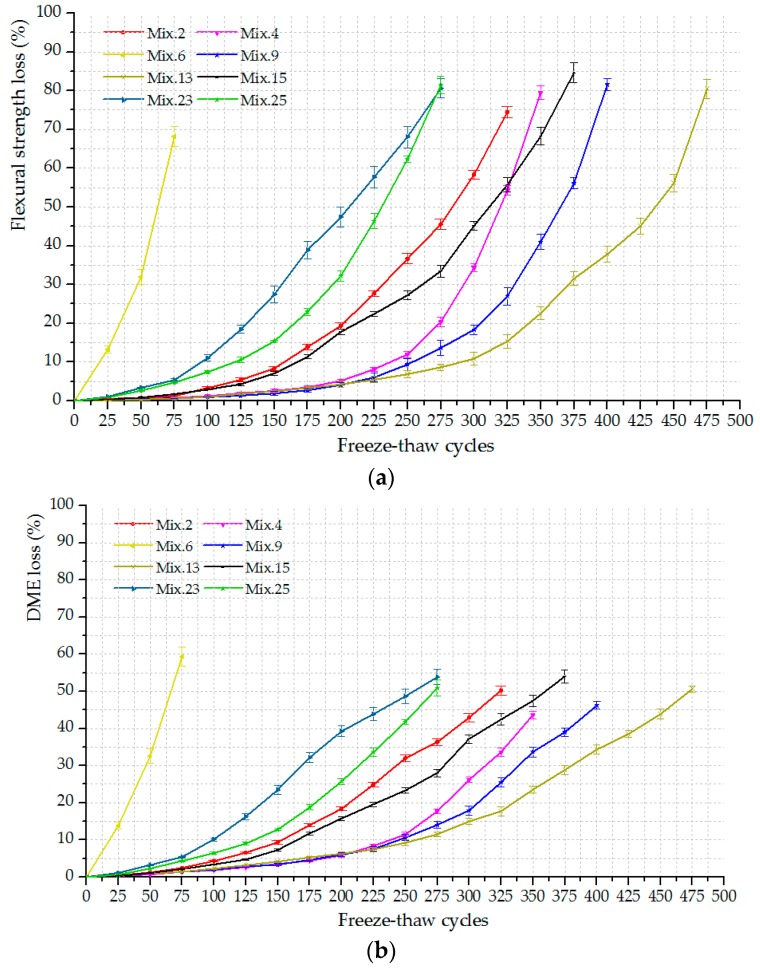
The engineering property loss of typical ECC mixtures versus freeze-thaw cycles (L-C curves): (**a**) The loss in flexural strength; (**b**) The loss in DME.

The DME and flexural strength were measured at each step (every 25 cycles) and the loss rates were calculated to plot their dependence on the freeze-thaw cycles (L-C curve), as presented in Figure 8. The loss in these material properties increases with the freeze-thaw process, which is treated as the working state of ECC mixtures. The ultimate freeze-thaw bearing capacity of each ECC mixture, expressed by the maximum bearable cycles, is recorded at the end of the freeze-thaw test. It is observed that Mix. 2, 4, 9, 13 and 15 with various volume fractions of PVA fiber reinforcement provide the outstanding resistance to the freeze-thaw exposure, since their engineering properties evaluated herein lose out slowly and the ultimate failure occurs after 300 cycles at least. On the other hand, the mixtures without fibers (Mix. 6) exhibit sharp reductions in the flexural strength and DME with the ascending freeze-thaw cycles.

### 3.3. The Identification of Frost-Induced Failure using the M-K Criterion

The ultimate failure (UF) for each mixture represents that the ECC materials are not effective any more. However, the behavior characteristics before UF are of greater concern in this investigation as some unseen features could exist during the frost-induced failure evolution. The M-K criterion was introduced to gain insight into the freeze-thaw working state leap of ECC through the *L-C* curve. This non-parametric statistical test is insensitive to outliers and regardless of data distributions [32,33,34]. By examining the sign of all pairwise differences of observed values, the trend in time series are quantified and significant mutational changes are identified. The M-K criterion has been proven to be a powerful tool in climatology [35], meteorology [36], hydrology [37] and the field of construction [38], providing useful information on the possibility to broaden its application to the detections of other trends. As shown in Figure 8, the loss in engineering properties has a temporal variation trend which embodies the working state of ECC materials. With these considerations, the M-K criterion is suitable for detecting the mutation of the working behavior of ECC materials under the freezing and thawing influence. In the application of the M-K criterion, a new stochastic variable *d_k_* at the *k*th step of measurement can be defined by,

(1) $$d_{k}=\sum_{i=1}^{k}m_{i}\left(2\leq k\leq n\right),m_{i}=\left\{ \begin{array}{ll} +1, & L\left(i\right)>L\left(j\right)\left(1\leq j\leq i\right)\\ 0, & otherwise \end{array}\right.$$

where *m_i_* is the cumulative number of the samples; “+1” means adding one more to the existing value if the inequality on the right side is satisfied for the *j*th comparison. {*L*(*i*)} is the sequence of the performance loss (flexural strength loss/DME loss) at *i*th step of measurement, which is consistent with the *L-C* curve. The mean value E(*d_k_*) and variance Var(*d_k_*) of *d_k_* can be calculated by,

(2) $$\begin{array}{l} E(d_{k})=k(k-1)/4\;(2\leq k\leq n),\\ \mathrm{Var}(d_{k})=k(k-1)(2k+5)/72\;(2\leq k\leq n) \end{array}$$

Assuming that the {*L*(*i*)} sequence is statistically independent, a new statistic *UF_k_* is defined by,

(3) $$UF_{k}=\left\{ \begin{array}{ll} 0, & k=1\\ (d_{k}-E(d_{k}))/\sqrt{\mathrm{Var}(d_{k})}, & 2\leq k\leq n \end{array}\right.$$

Thus, an *UF_k_-C* curve can be formed using the *UF_k_* data. A similar procedure proceeds the inverse {*L*(*i*)} sequence, which is denoted by {*L*′(*i*)} as follow,

(4) $$L(i)=L^{\prime }(n-i+1)$$

where *n* is the sample capacity. Similarly, the stochastic variable *d*′_*k*_ at the *k*th step of measurement is defined by,

(5) $${d^{\prime }}_{k}=\sum_{i=1}^{k}m_{i}\left(2\leq k\leq n\right),m_{i}=\left\{ \begin{array}{ll} +1, & L^{\prime }\left(i\right)>L^{\prime }\left(j\right)\left(1\leq j\leq i\right)\\ 0, & otherwise \end{array}\right.$$

where *m_i_* is also the cumulative number of the samples; “+1” means adding one more to the existing value if the inequality on the right side is satisfied for the *j*th comparison. The mean value E(*d*′_*k*_) and the variance Var(*d*′_*k*_) of *d*′_*k*_ can be calculated following,

(6) $$\begin{array}{l} E({d^{\prime }}_{k})=k(k-1)/4\;(2\leq k\leq n),\\ \mathrm{Var}({d^{\prime }}_{k})=k(k-1)(2k+5)/72\;(2\leq k\leq n) \end{array}$$

Here, *d*′_*k*_ represents the degree of the ascending trend of the {*L*′(*i*)} sequence. It is important to note that the inversed sequence has a changing trend contrary to the original one. As a result, the trend of the inverse sequence should be correctly characterized by the opposite sign so that a new statistic *UB*′_*k*_ is defined by,

(7) $${{UB}^{\prime }}_{k}=\left\{ \begin{array}{cc} 0, & k=1\\ -({d^{\prime }}_{k}-E({d^{\prime }}_{k}))/\sqrt{\mathrm{Var}({d^{\prime }}_{k})}, & 2\leq k\leq n \end{array}\right.$$

and the statistic *UB_k_* corresponding to the original step of measurement can be calculated by,

(8) $$UB_{k}=U{B^{\prime }}_{n-k+1}$$

An *UB_k_-C* curve can be formed using the *UB_k_* data. The intersection of the *UF_k_-C* and *UB_k_-C* curves indicates the mutation point of the *L-C* curve. Consequently, the working state leaps of ECC materials can be distinguished following this criterion.

By distinguishing the intersections of *UF_k_* and *UB_k_* curves, the M-K criterion reveals two working state leaps for each ECC mixtures. These two significant mutational changes characterize the updated failure induced by a certain number of freeze-thaw cycles. The first mutational change is defined as the initial frost-induced failure (IF), which can be identified by investigating the whole *L-C* curve. Then, the following mutational change identified from the *L-C* curve segment (from IF on) is defined as the progressive frost-induced failure (PF). The IF and PF indices are the completed freeze-thaw cycles at specific working state leap. The results are summarized in Table 4 together with the UF indices (the terminal freeze-thaw cycles). For each ECC mixture, the IF and PF indices are determined based on the loss data of flexural strength and then validated by those of the DME. Identical results are obtained from the different data series. It should be noted that such a determination of IF and PF indices is an estimation of the real value because the loss data is collected discontinuously after every 25 cycles.

### 3.4. The Characterization of Freeze-Thaw Stages

**Figure 9:**
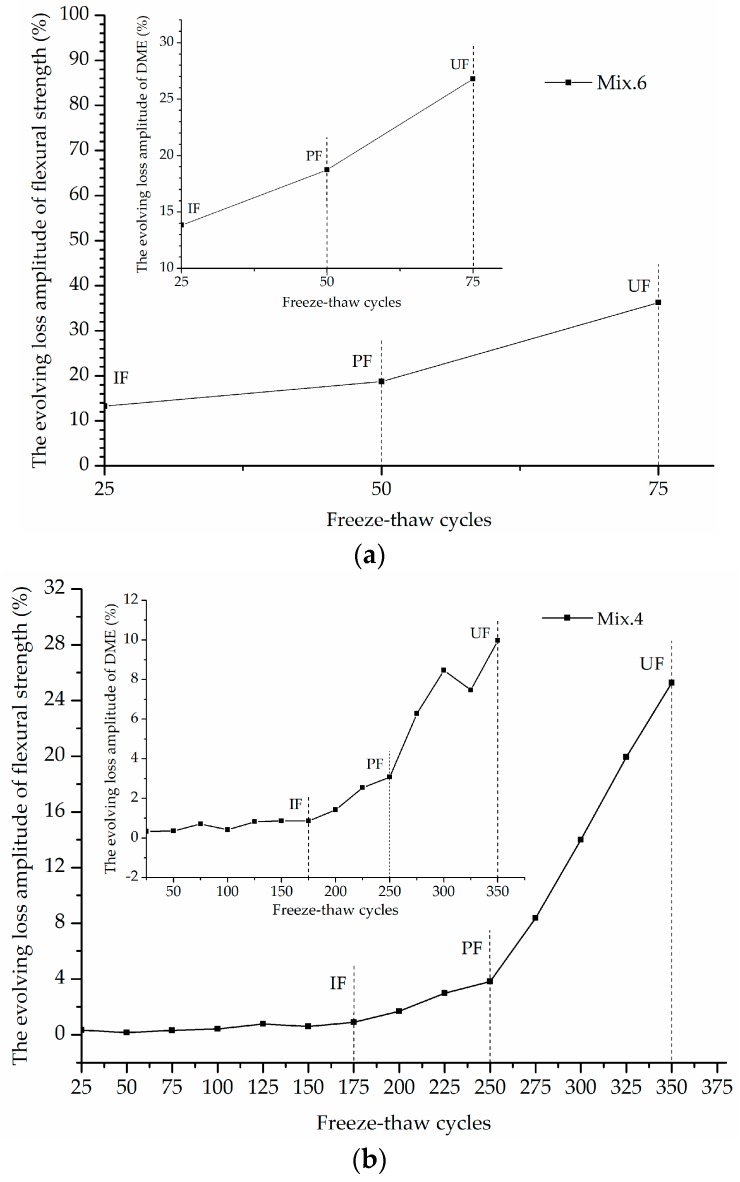

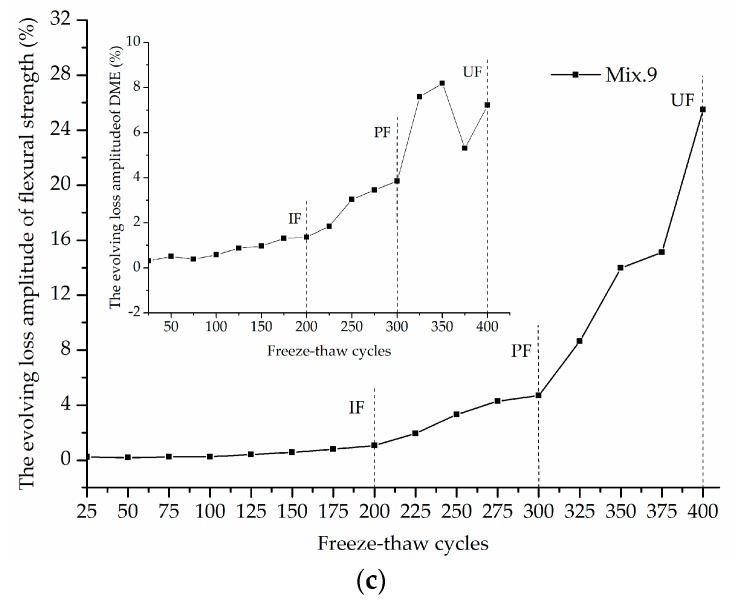
The evolving loss amplitude of engineering property for typical ECC mixtures: (**a**) Mix. 6; (**b**) Mix. 4; (**c**) Mix. 9.

**Figure 10:**
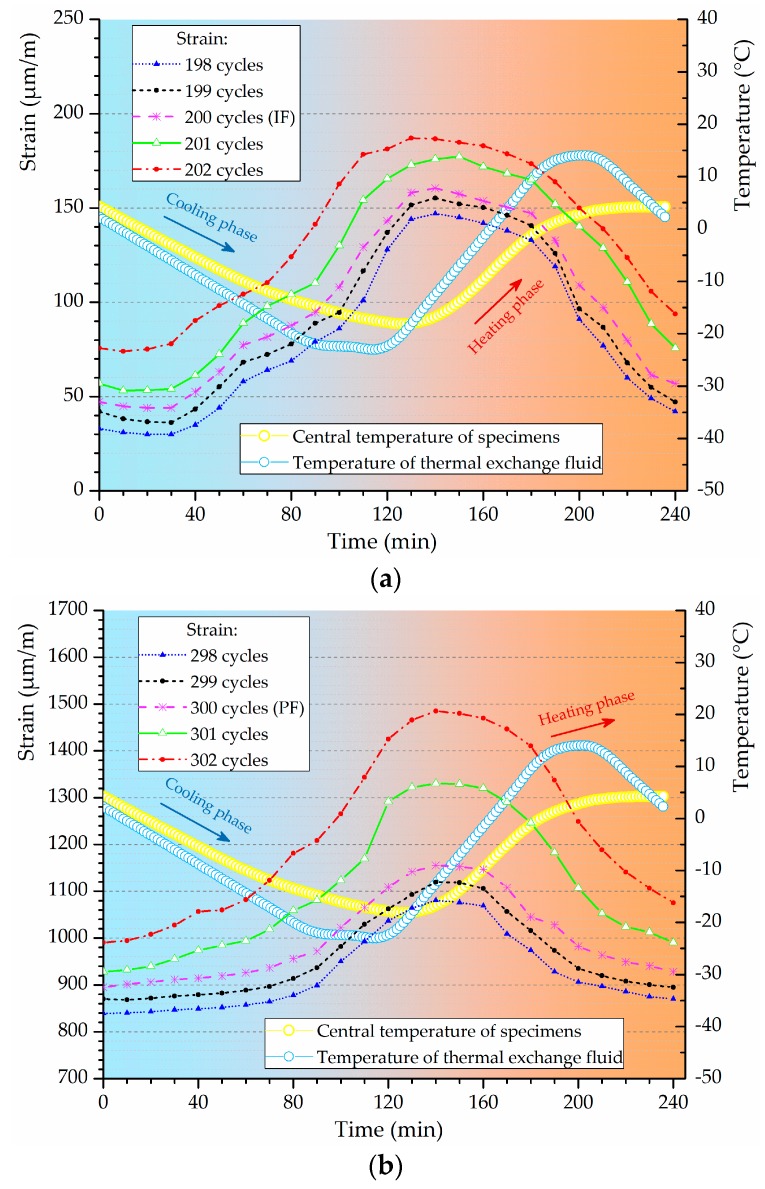

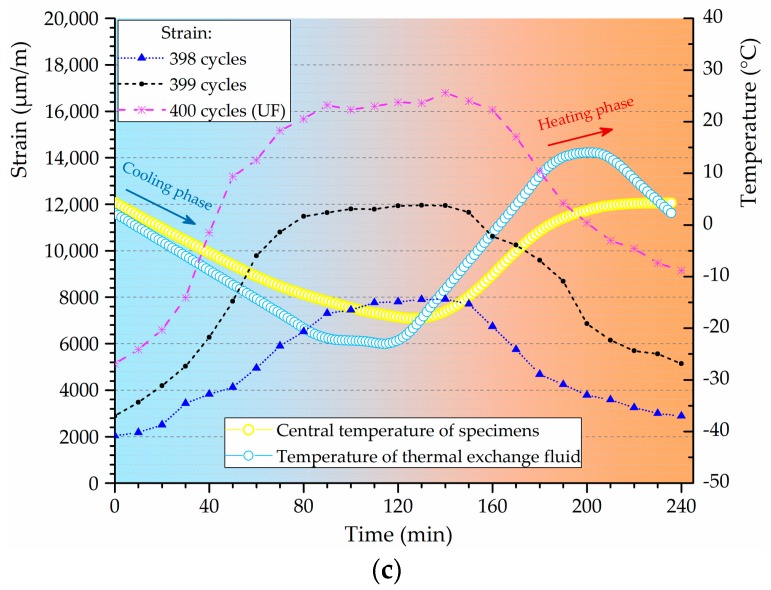
The strain variations of Mix. 9 with temperature profile under freeze-thaw cycles around different failure point: (**a**) IF; (**b**) PF; (**c**) UF.

**Figure 11:**
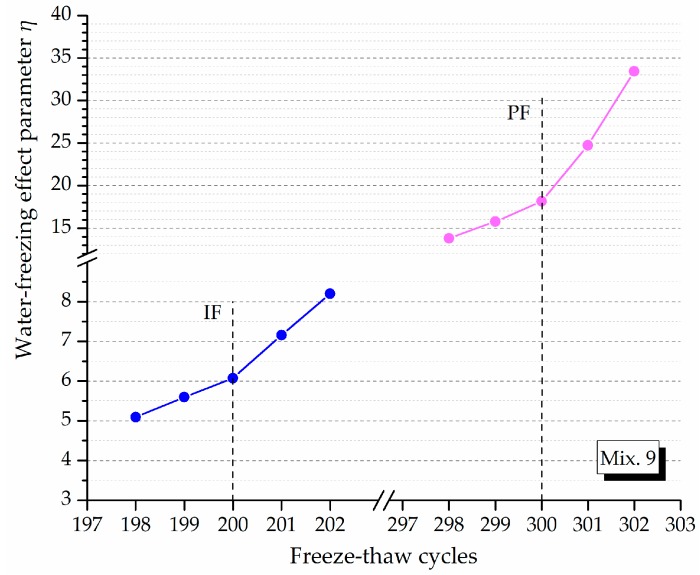
The water-freezing effect parameter *η* versus the freeze-thaw cycles.

The IF and PF identified using M-K criterion are plotted in the evolving loss amplitude of flexural strength and DME of typical ECC mixtures, as shown in Figure 9. The strain variation curves of Mix. 9 under freeze-thaw cycles around the frost-induced failure points are shown in Figure 10. Each of the freeze-thaw cycles is divided into cooling and heating phase according to the different temperature variance tendency. The water transformation into ice accompanied by a volume expansion causes the hydraulic pressure in the cooling phase [39,40]. Besides, the osmotic pressure is built up as the response to the movement of supercooled gel water from the non-frozen sites to the ice bodies [41,42]. These freezing-induced pressures are among the driving forces for the volumetric change gauged inside the specimen. Assuming that all the pores are filled with water, the volumetric change is mostly related to the freezable water quantities. The water-freezing effect parameter *η* is proposed herein to model the volumetric change of ECC specimen in a single freeze-thaw cycle, which is formulated by time integration at a volume unit:

(9) $$\eta =\frac{\int_{0}^{t}\varepsilon (t)dt}{V}$$

where *ε*(*t*) (μm/m) is the time-varying strain, *t* (min) is the time required for one cycle and *V* (cm^3^) is the volume of the ECC specimen. The results are illustrated in Figure 11 and the change of *η* value indicates that the content of water that can be frozen is different from the preceding cycles.

**Figure 12:**
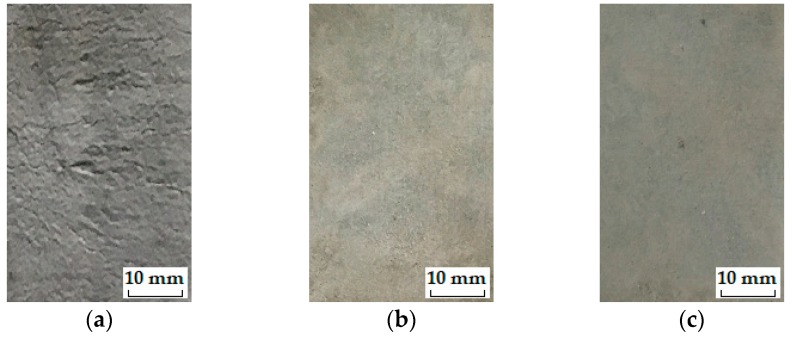
The surface appearance of typical ECC mixtures: (**a**) Mix. 6 after 25 cycles; (**b**) Mix. 4 after 175 cycles; (**c**) Mix. 9 after 200 cycles.

**Figure 13:**
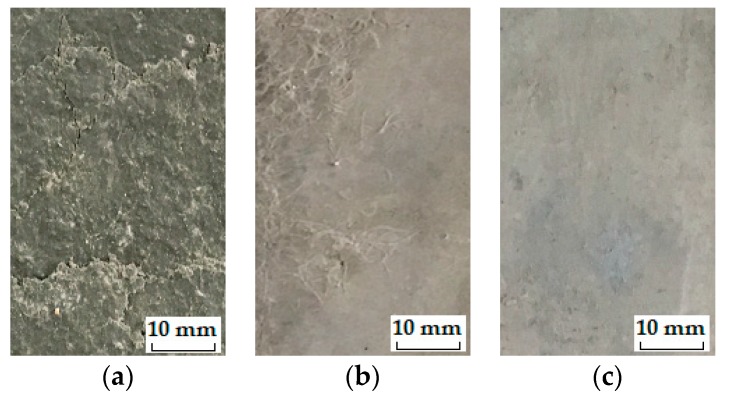
The surface appearance of typical ECC mixtures: (**a**) Mix. 6 after 50 cycles; (**b**) Mix. 4 after 250 cycles; (**c**) Mix. 9 after 300 cycles.

**Figure 14:**
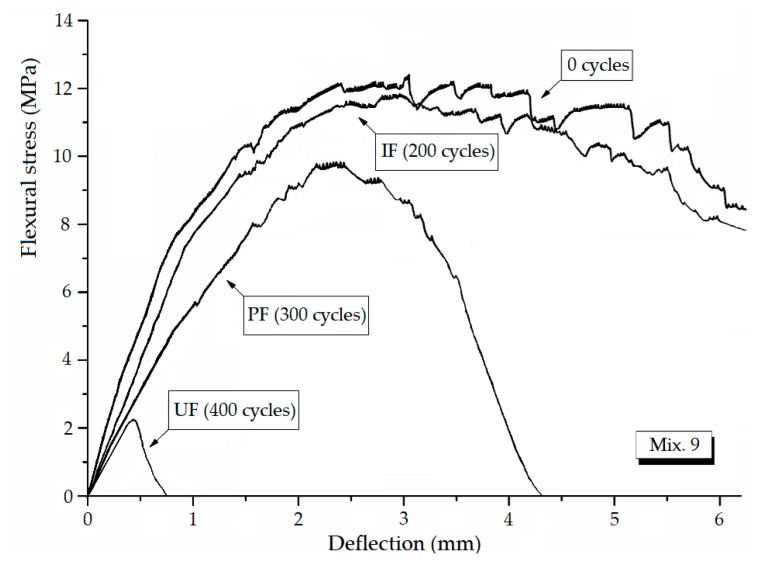
The changing flexural behavior of typical ECC mixture.

**Figure 15:**
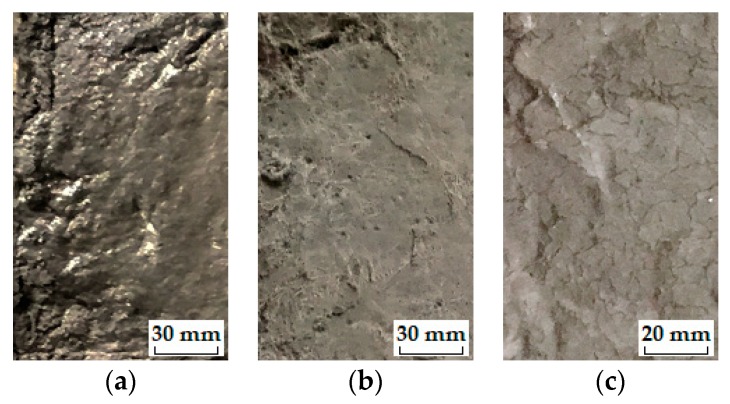
The surface appearance of typical ECC mixtures: (**a**) Mix. 6 after 75 cycles; (**b**) Mix. 4 after 350 cycles; (**c**) Mix. 9 after 400 cycles.

The updated failure concept characterizes three different stages of ECC mixtures over their freeze-thaw working state process:

1. The stable stage. As shown in Figure 9, the loss in DME and flexural strength grows at quite a slow pace and the developing trend is steady before the *IF*, representing a stable stage under the influence of freeze-thaw cycles. During the heating phase, the freezing-induced pressures are being relieved therefore the deformation strain is generally reduced. However, the volumetric change has become plastic after certain cycles of freeze and thaw so that the strain is not able to return to the beginning. This particular strain characteristic could be found in Figure 10a where the strain variation paths of Mix. 9 are not overlapped at the approach of *IF*. Actually, the residual strain is generated, due to the irreversible influence of freeze-thaw action. The residual strain keeps increasing and the mutation of strain variation happens in the wake of *IF*. The value of water-freezing effect parameter *η* leaps synchronously, as shown in Figure 11, indicating the content of freezable water is significantly increased from then on. The most possible reason is the initiation of micro-cracks at the *IF*. Since the flexural strength and DME are sensitive to the micro-cracking, the sudden change in the evolving loss amplitude of engineering properties is observed directly following the stable stage, as shown in Figure 9. In addition, the ECC mixtures without fibers (Mix. 6) suffer some scaling-off on the surface at the *IF*, which is not observed on the fibrous ones (Mix. 4 and 9), as shown in Figure 12. Normally the plain specimen is cast with more inherent defects, hence spalling is very likely to occur even if the freezing-induced pressures have not been that influential.
2. The unstable stage. After the *IF*, the ECC mixtures experience the greater evolving loss in engineering properties when compared with that in the stable stage, as shown in Figure 9. Actually, the ECC materials have transited into a stage that is relatively unstable. The residual strain, shown in Figure 10b, is evidently greater than that of the stable stage, indicating the propagation of micro-cracks. With continuous water supply, the freezing-induced pressures are magnified, and the strain variation mutates immediately after the PF, as shown in Figure 10b. Meanwhile, another inflexion point for the *η* value is found at the PF, as shown in Figure 11. Moreover, the surface scaling is observed on the ECC mixtures reinforced with PVA fibers at the PF, as shown in Figure 13b,c, yet less noticeable than those prepared without fiber reinforcement, as shown in Figure 13a. It implicates that some of the micro-cracks have been connected into networks, thereby forming the macro-cracks. Accordingly, the obvious changes in flexural behavior are found at the end of this stage. The flexural strength decreases, and the energy dissipation capacity is weakened, as shown in Figure 14.
3. The yield stage. It can be seen from Figure 9 that the loss in engineering properties sharply increases after the progressive frost-induced failure, implying that these ECC mixtures go into the yield stage totally different from the previous two. The performance of the ECC materials is declining, due to the massive frost damage during this stage. With the development of freeze-thaw damage, the ECC materials become increasingly porous and permeable. More free water penetrates into the pores and cracks, intensifying the freezing expansion until the occurrence of ultimate failure, as shown in Figure 15. The freezing-induced pressure is especially great in the later period of yield stage, which makes the ECC matrix produce considerable deformation strain, as shown in Figure 10c. At the end of this stage, the flexural performance is badly impaired, and the behavior of brittleness is exhibited under the bending load, as shown in Figure 14.

The ECC’s three-stage working behavior revealed above characterizes the gradual failure process and vividly embodies the essence in the ECC failure evolution under the freeze-thaw influence. Frost-induced failure primarily take place inside the ECC specimens and gradually extend to the exterior (i.e., from micro-cracks to macro-cracks, and finally the destruction). The development of flexural performance loss, DME loss, internal strain and water-freezing effect parameter through different stages reflect the extent to which the freeze-thaw cycles affect the ECC materials. 

It can be stated that before and after each individual frost-induced failure point, these ECC mixtures under a certain cyclic freeze-thaw case could belong to different categories with different material characteristics. In this regard, the ECC materials are subjected to impressive damage at the two points, so that the material properties and working behavior have changed in essence for the different stages. The characteristic of ECC materials working in the unstable stage is the micro-cracking at the first failure point and the growth of micro-cracks afterwards. The ECC materials with micro-cracks can still withstand freeze-thaw damage and the span of the unstable stage is extended because of the effectiveness of PVA fiber reinforcement. All these facts are the reflection of the generalized ‘toughness’ brought about by PVA fibers under the influence of freezing and thawing. 

Furthermore, these two updated frost-induced failure indices (IF and PF index), along with the UF index, can be developed into a three-level evaluation model assisting the anti-frost design for ECC materials, as well as other cement-based materials. Under this mode, the materials are designed orienting the requirement from a certain level (IF, PF or UF index) depending on their specific use. In other words, the materials prepared for the crucial civil infrastructures are highly recommended to be designed using the IF index as the control level. And the PF index can be employed as the control level for the civil infrastructures of less importance whilst the UF index can be used for common constructions.

### 3.5. Influence of Multi-Factors on the Performance Characteristics

It is necessary to study how the flexural strength and frost-induced failure indices are affected by different mix design factors. The statistical performance measure of signal-to-noise (S/N) ratio, instead of the average observed values, has been employed to interpret the test data into a value deemed to be the quality response. The S/N ratio is transferred from the loss function, which is introduced to calculate the deviations between the test value and the desired value to evaluate each independent factor or their interaction on the evaluated performance [43]. The Taguchi method typically provides the S/N ratio with three varieties of quality responses available [44]. Given that the maximum responses (flexural strength and frost-induced failure indices) are desired, the larger-the-better quality response is applicable for the performance characteristics evaluated herein. The S/N ratio is logarithmically formulated as,

(10) $$S/N=-10\times \log_{10}(\frac{1}{n}\sum_{i=1}^{n}\frac{1}{y_{i}^{2}})$$

where, *n* indicates the number of observations and *y* represents the results of the measurement.

**Figure 16:**
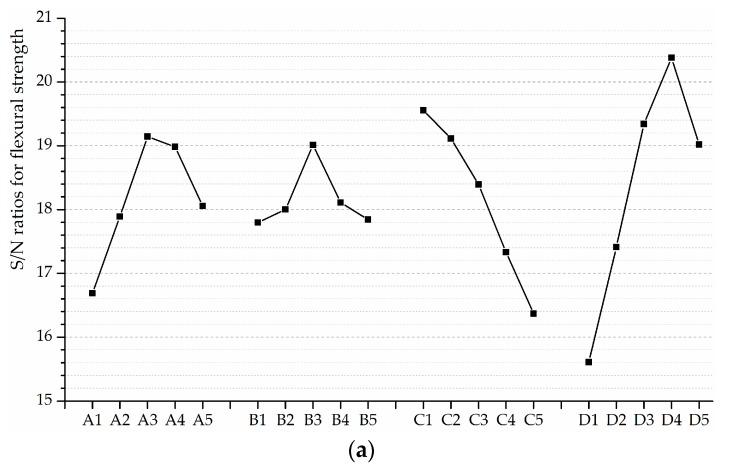

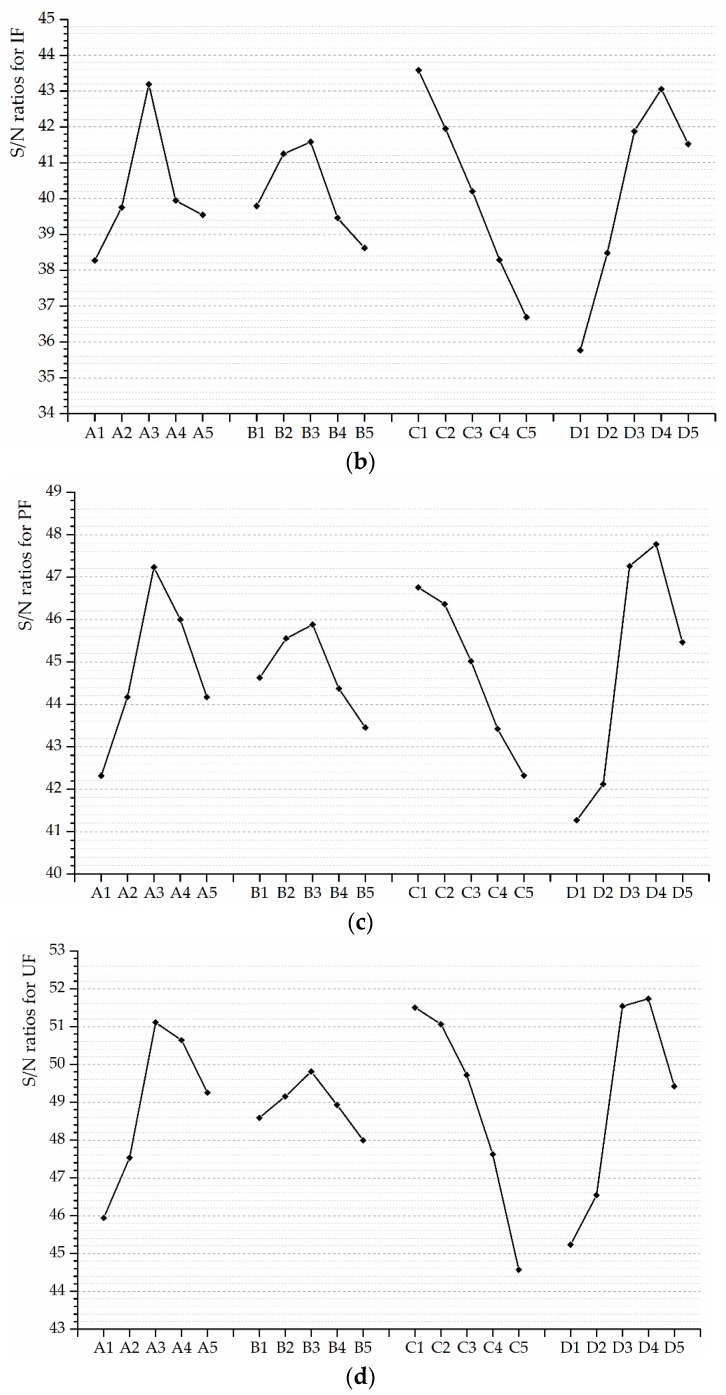
S/N ratios for: (**a**) Flexural strength; (**b**) IF index; (**c**) PF index; (**d**) UF index.

As shown in Figure 16a, the higher *W*/*B* has a negative effect on the flexural strength. The flexural strength of ECC is enhanced by 0.5~2 vol.% of the PVA fiber reinforcement. It is attributed to the micro-cracking resisting effect of PVA fibers. Nevertheless, there is a tendency of strength degradation resulting from the blending of fibers. Excessive fibers are likely to worsen the density of specimens, which could interpret the decrease in the flexural strength occurs at the *V_PVA_* beyond 1.5%. Besides, the flexural strength of ECC can obviously benefit from a moderate level of *FA* (around 0.35) and *S*/*B* (around 0.5).

The dependences of these frost-induced failure indices on various factors are plotted in Figure 16b–d. It is observed that all the indices decrease with the increasing of *W*/*B*. The more water added during the mixing procedure leads to the earlier occurrence of frost-induced failure, as the porosity characteristics is worse. The promotion to the freeze-thaw resistance is found in the presence of PVA fibers with the volume fraction between 0.5~2.0%. The updated frost-induced failure and the ultimate failure are delayed substantially as a result of PVA fiber reinforcement. The air-entraining and pressure-release effect of PVA fibers facilitate ECC materials to slow the formation of micro-cracks and work in the stable stage for a relatively long period. Even in the unstable stage, the micro-cracks can be bridged by PVA fibers as an effective way to mitigate crack growth. However, a small reduction in the indices is observed in ECC with 2.0 vol.% of PVA fiber reinforcement. It could be explained that excessive fibers are detrimental to their dispersion in fresh cementitious composites, which impairs the density of hardened materials.

As shown in Figure 16b–d, the substitution of fly ash up to 35% is the most beneficial to the freeze-thaw resistance. The pozzolanic reaction between fly ash and calcium hydroxide generated from the hydration of cement results in the improvement in porosity characteristics. Additional fly ash is consumed by the pozzolanic reaction with aging, which helps to reduce the size and amounts of capillary pores. Consequently, the obvious rise in PF and UF indices is found at *FA* = 0.525, as shown in Figure 16c,d. However, the calcium hydroxide would become insufficient for the pozzolanic reaction if too much cement is replaced by the fly ash. That is why the decline of indices is observed at *FA* = 0.7. Moreover, reasonable sand content favors the workability of fresh cementitious composites so that adequate density and strength can be gained before the frost exposure. However, the increase of *S*/*B* simultaneously decreases the paste ratio, thereby leading to the decline in failure indices, as shown in Figure 16b,d.

The higher frost-induced failure index indicates the later occurrence of frost-induced failure, i.e., the better freeze-thaw resistance. The optimum mix formulation is deemed to be reachable where each individual factor achieves the highest frost-induced failure indices, namely, to unite the levels of the highest S/N ratios. Therefore, the optimum freeze-thaw resistance mix formulation recommended in this investigation is: A3B3C1D4 (*FA*: 35.0%, *S*/*B*: 0.5, *W*/*B*: 0.25, *V_PVA_*: 1.5%). This mix formulation is estimated to provide the optimum resistance against frost-induced failure because of the highest IF, PF and UF indices. Besides, some parameters are of little benefit to the performance characteristic (e.g., the 0.25 for *S*/*B*, 0.5 for *W*/*B*, 0 and 0.005 for *V_PVA_*) on account of their low S/N ratios. They are consequently not the ideal selection for the mix design of ECC materials.

### 3.6. Analysis of Variance

The analysis of variance (ANOVA) is used to perceive the relative importance of each mix design factor. This approach defines *DF* as the degree of freedom and *SS* as the sum of squares for a given variable. Therefore, the mean square (*MS*) is formulated as,

(11) $$MS=\frac{SS}{DF}$$

The statistical method of F-test is applied to present the significance of each factor. The statistic *F*-value can be calculated as follow,

(12) $$F=\frac{MS_{f}}{MS_{e}}$$

where *MS_f_* and *MS_e_* are the mean square (variance) for the individual factor and error, respectively. Therefore, the *p*-value is determined by,

(13) $$p=P(F,DF_{f},DF_{e})$$

where *DF_f_* and *DF_e_* are the degree of freedom for the individual factor and error, respectively. The ANOVA is implemented by Minitab 17 and the results are summarized in Table 5, Table 6, Table 7 and Table 8 using the 95% confidence level. The symbol “O” and “X” denote the significant and insignificant factor, respectively.

Based on the analysis of variance, *FA*, *W*/*B* and *V_PVA_* are identified as the significant factors for raising the flexural strength and frost-induced failure indices. The influence of *S*/*B* is found to be insignificant, as its *F*-values are lower than the critical value (3.84) and *p*-values are lower than 0.05. As the higher *F*-value represents the greater significance, the ranking of the mix design factors for the flexural strength, IF and PF indices is: *V_PVA_* > *W*/*B* > *FA* > *S*/*B*, and *W*/*B* > *V_PVA_* > *FA* > *S*/*B* for the UF index.

**Figure 17:**
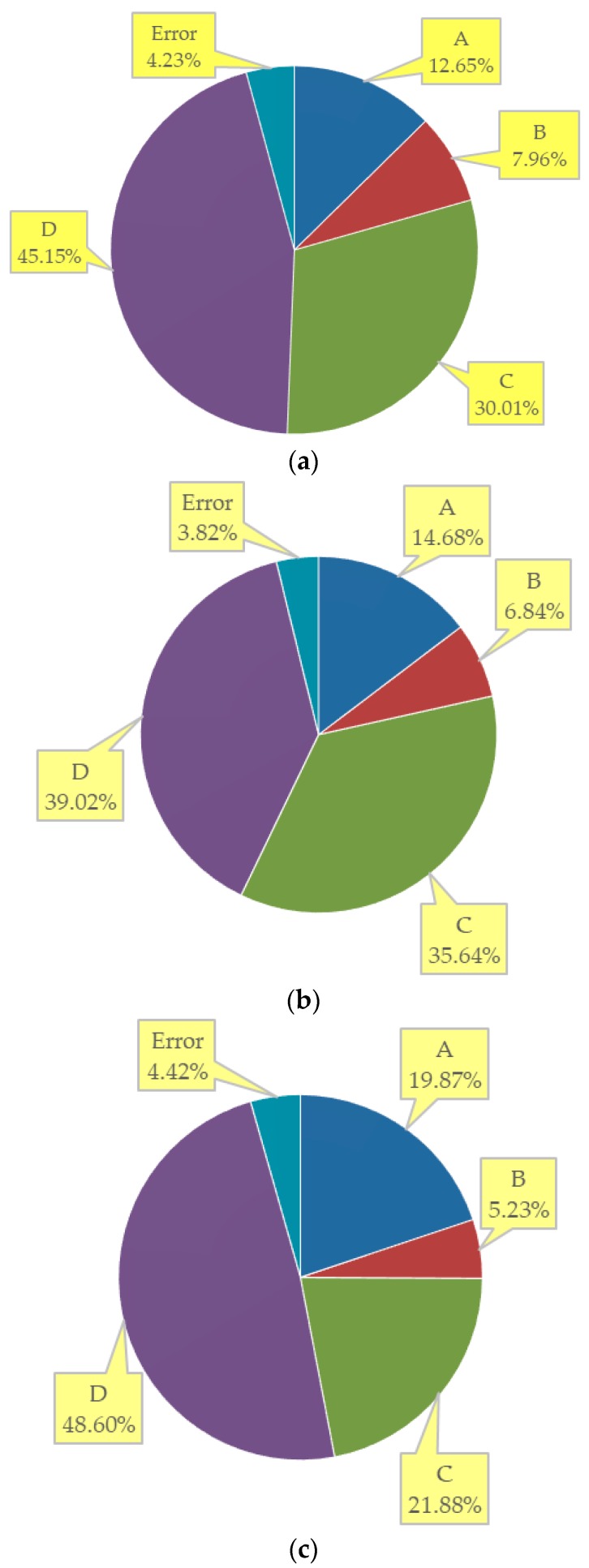

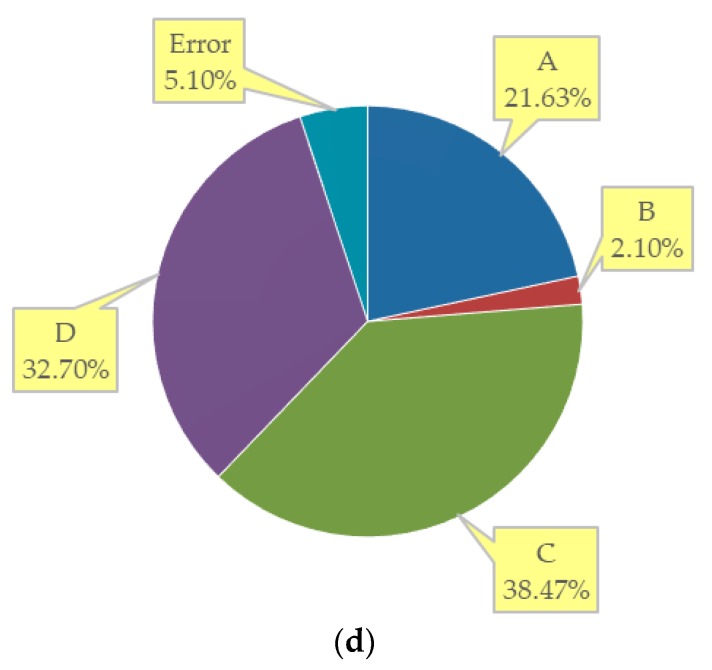
Distribution of contribution ratio for: (**a**) Flexural strength; (**b**) IF index; (**c**) PF index; (**d**) UF index.

Figure 17 presents the contribution ratio of each factor individually. The *V_PVA_* makes the most contribution to the flexural strength as expected (45.15%), followed is the *W*/*B* (30.01%). Either of the two factors provides the ratio exceeding the sum of *FA* and *S*/*B* (20.61%). In terms of the IF index, the *V_PVA_* is observed to account for the highest percentages underneath the total contributions which achieves 39.02%. Furthermore, the contribution of PVA fiber reinforcement to resist the PF is more impressive (48.60%), implying the merits of PVA fibers as an effective reinforcement system for enhancing the freeze-thaw “toughness” of the ECC materials. The *W*/*B* contributes 38.47% to the UF index, more than that obtained with any other factors. The contribution ratios of *FA* are 14.68%, 19.87% and 21.63% respectively, implying that finer fractions of the fly ash are conducive to the freeze-thaw resistance. It could be attributed to the relatively significant effect of water-to-binder ratio, the volume fraction of PVA fibers and ground fly ash content on the development of strength and density prior to the frost exposure.

### 3.7. Confirmation Experiment

Since the optimum mix formulation (A3B3C1D4) inferred from the analysis on the influence of multi-factors is not included in the *L*_25_ orthogonal array, it is necessary to confirm the actual values of frost-induced failure indices for this formulation. Specimens are prepared following the combination of *FA* (35%), *S*/*B* (0.5), *W*/*B* (0.25) and *V_PVA_* (1.5%), and then subjected to the rapid freeze-thaw test. The tests of fundamental transverse and flexural strength are conducted after every 25 cycles. The results regarding the loss rates of DME and flexural strength are used in the trend analysis by the M-K criterion. The tested values for the IF and PF indices are thus determined. Meanwhile, the frost-induced failure index for the mix formulation (A3B3C1D4) is estimated by,

(14) $$F_{E}=\bar{F}+({\bar{A}}_{3}-\bar{F})+({\bar{B}}_{3}-\bar{F})+({\bar{C}}_{1}-\bar{F})+({\bar{D}}_{4}-\bar{F})$$

where $\bar{F}$ is the average value of the failure index; ${\bar{A}}_{3}$, ${\bar{B}}_{3}$, ${\bar{C}}_{1}$ and ${\bar{D}}_{4}$ are the means of failure indices corresponding to mix design factors at their respective optimal levels; *F_E_* denotes the estimated value of the failure index. With the reliability of the condition assumed to be 95%, the confidence interval can be calculated, as follows [45],

(15) $$CI_{CE}=[F_{0.05}(1,f_{e})V_{e}((1+T_{\mathrm{dof}})/N+(1/S)){]}^{\left(1/2\right)}$$

where *F*_0.05_(1, *f*_e_) is the *F*-value for the confidence interval of 95% with *f*_e_ referring to the errors’ freedom degree; *V*_e_ is the error variance; *S* is the number of replications for confirmation experiments; *N* is the total number of experiments; *T_dof_* is the total degrees of freedom associated with the estimated value. 

The experimental and estimated values for the frost-induced indices are summarized in Table 9. It is evident that the experimental values are well within the 95% confidence interval.

## 4. Conclusions

This paper innovatively studies the working behavior of the ECC materials subjected to frost exposure by introducing the concept of freeze-thaw working state and the M-K criterion. The essential failure characteristics of ECC materials are revealed from the beginning to the end of repeated freeze-thaw action. The analytical results draw the following conclusions.

1. The M-K criterion can distinguish the ECC mixtures’ freeze-thaw working state leaps, which is verified by the incremental amplitude analysis on the loss trends of flexural performance and DME, the strain variation behavior, the water-freezing effect parameter, as well as the surface examination. The essential leap feature is an objective and essential attribute of materials complying with the natural course from quantitative change to qualitative change. Hence the attribute rationally updates the definition of the existing frost-induced failure.
2. It is revealed that the ECC materials during the whole freeze-thaw process have three different working stages, leading to the definitions of the initial frost-induced failure (*IF*) and progressive frost-induced failure (*PF*), respectively. IF is the critical point of freeze-thaw working state between being stable and unstable. PF is the other transition point after *IF*. The ECC materials rapidly become weaker and closer to the ultimate failure from PF on. It does not necessarily mean that the ECC materials break down at the updated failure point. Actually, the ECC materials working in the unstable and yield stages can still withstand repetitive freeze-thaw actions despite the growth of damage and the evolution of material characteristics. Based on the updated failure concept, the three-level evaluation model is proposed to complement the existing anti-frost design method.
3. The proper selection of volume fraction makes PVA fibers of significant value towards improving the ability of ECC materials to resist frost-induced failure. The span of the freeze-thaw stage can be substantially extended before the occurrence of ultimate failure, which demonstrates the desirable frost durability provided by PVA fiber reinforcement.
4. The ground fly ash with finer particle sizes than original ones is verified to be effective in improving the resistance against frost-induced failure for the ECC materials, from the premise that the content of ground fly ash is right for the pozzolanic reaction.

## Figures and Tables

**Table 1:** The chemical compositions of binders.

Chemical Analysis Basic Oxides (%)	Portland Cement	Ground Fly Ash
SiO_2_	21.08	55.70
Al_2_O_3_	5.47	25.63
Fe_2_O_3_	3.96	5.65
CaO	62.28	6.93
MgO	1.73	2.25
SO_3_	2.63	0.58
R_2_O	0.50	0.60

**Table 2:** The properties of polyvinyl alcohol (PVA) fibers.

Fiber	Length (mm)	Diameter (μm)	Density (g/cm^3^)	Tensile Strength (GPa)	Elastic Modulus (GPa)	Elongation (%)
PVA	13	39	1.3	1.6	42	7

**Table 3:** The mix formulations for engineered cementitious composites (ECC) using *L*_25_ orthogonal array.

Mixture	Labels	Combinations				Superplasticizer (%)
		A(*FA*)	B(*S*/*B*)	C(*W*/*B*)	D(*V_PVA_*)	
1	A1B1C1D1	0	0.250	0.2500	0	0.82
2	A1B4C3D2	0	0.625	0.3750	0.005	0.38
3	A1B2C5D3	0	0.375	0.5000	0.010	0.09
4	A1B5C2D4	0	0.750	0.3125	0.015	1.07
5	A1B3C4D5	0	0.500	0.4375	0.020	0.94
6	A2B4C2D1	0.175	0.625	0.3125	0	0.22
7	A2B2C4D2	0.175	0.375	0.4375	0.005	0.05
8	A2B5C1D3	0.175	0.750	0.2500	0.010	1.51
9	A2B3C3D4	0.175	0.500	0.3750	0.015	0.30
10	A2B1C5D5	0.175	0.250	0.5000	0.020	0.50
11	A3B2C3D1	0.350	0.375	0.3750	0	0.63
12	A3B5C5D2	0.350	0.750	0.5000	0.005	0.06
13	A3B3C2D3	0.350	0.500	0.3125	0.010	0.48
14	A3B1C4D4	0.350	0.250	0.4375	0.015	0
15	A3B4C1D5	0.350	0.625	0.2500	0.020	1.52
16	A4B5C4D1	0.525	0.750	0.4375	0	0.48
17	A4B3C1D2	0.525	0.500	0.2500	0.005	0.68
18	A4B1C3D3	0.525	0.250	0.3750	0.010	0.10
19	A4B4C5D4	0.525	0.625	0.5000	0.015	0.05
20	A4B2C2D5	0.525	0.375	0.3125	0.020	0.59
21	A5B3C5D1	0.700	0.500	0.5000	0	0
22	A5B1C2D2	0.700	0.250	0.3125	0.005	0.67
23	A5B4C4D3	0.700	0.625	0.4375	0.010	0.04
24	A5B2C1D4	0.700	0.375	0.2500	0.015	1.42
25	A5B5C3D5	0.700	0.750	0.3750	0.020	0.87

**Table 4:** The frost-induced failure indices for ECC mixtures.

Mixtures	IF Index	PF Index	UF Index
1	25	50	75
2	150	225	325
3	150	225	325
4	175	250	350
5	150	225	300
6	25	50	75
7	100	150	200
8	175	275	375
9	200	300	400
10	125	200	275
11	75	125	175
12	100	150	200
13	225	350	475
14	150	225	325
15	175	275	375
16	25	50	75
17	175	250	350
18	150	225	325
19	150	225	300
20	175	250	350
21	25	50	75
22	150	225	300
23	125	200	275
24	175	275	375
25	125	200	275

**Table 5:** Results of ANOVA for flexural strength.

Factor	*DF*	*SS*	*MS*	*F*	*p*	Contribution (%)	Significance
A	4	18.027	4.51	5.98	0.0158	12.65	O
B	4	11.335	2.83	3.76	0.0424	7.96	X
C	4	42.765	10.69	14.19	0.0010	30.01	O
D	4	64.333	16.08	21.35	0.0003	45.15	O
Error	8	6.027	0.75	-	-	4.23	-
Total	24	142.487	-	-	-	100.00	-

**Table 6:** Results of ANOVA for IF index.

Factor	*DF*	*SS*	*MS*	*F*	*p*	Contribution (%)	Significance
A	4	66.44	16.61	7.69	0.0076	14.68	O
B	4	30.94	7.74	3.58	0.0589	6.84	X
C	4	161.29	40.32	18.66	0.0004	35.64	O
D	4	176.58	44.15	20.43	0.0003	39.02	O
Error	8	17.29	2.16	-	-	3.82	-
Total	24	452.540	-	-	-	100.00	-

**Table 7:** Results of ANOVA for PF index.

Factor	*DF*	*SS*	*MS*	*F*	*p*	Contribution (%)	Significance
A	4	71.5	17.88	8.98	0.0047	19.87	O
B	4	18.8	4.70	2.36	0.1399	5.23	X
C	4	78.73	19.68	9.89	0.0035	21.88	O
D	4	174.85	43.71	21.97	0.0002	48.60	O
Error	8	15.92	1.99	-	-	4.42	-
Total	24	359.80	-	-	-	100.00	-

**Table 8:** Results of ANOVA for UF index.

Factor	*DF*	*SS*	*MS*	*F*	*p*	Contribution (%)	Significance
A	4	93.48	23.37	8.48	0.0056	21.63	O
B	4	9.07	2.27	0.82	0.5459	2.10	X
C	4	166.30	41.57	15.09	0.0009	38.47	O
D	4	141.34	35.33	12.82	0.0015	32.70	O
Error	8	22.04	2.76	-	-	5.10	-
Total	24	432.23	-	-	-	100.00	-

**Table 9:** Confirmation test for the optimum level condition.

Optimum Mix Formulation (Mix. A3B3C1D4)	IF Index	PF Index	UF Index
Estimated values	222 ± 19.77	337 ± 44.84	456 ± 74.93
Experimental values	225	375	500
